# Supplementary figures and images for: Identification of an innexin required for termination of the asexual state in planarian reproductive switching
Source: PLoS Genet. 2025 Nov 18;21(11):e1011944. doi: 10.1371/journal.pgen.1011944 (PMC12654950; doi:10.1371/journal.pgen.1011944)

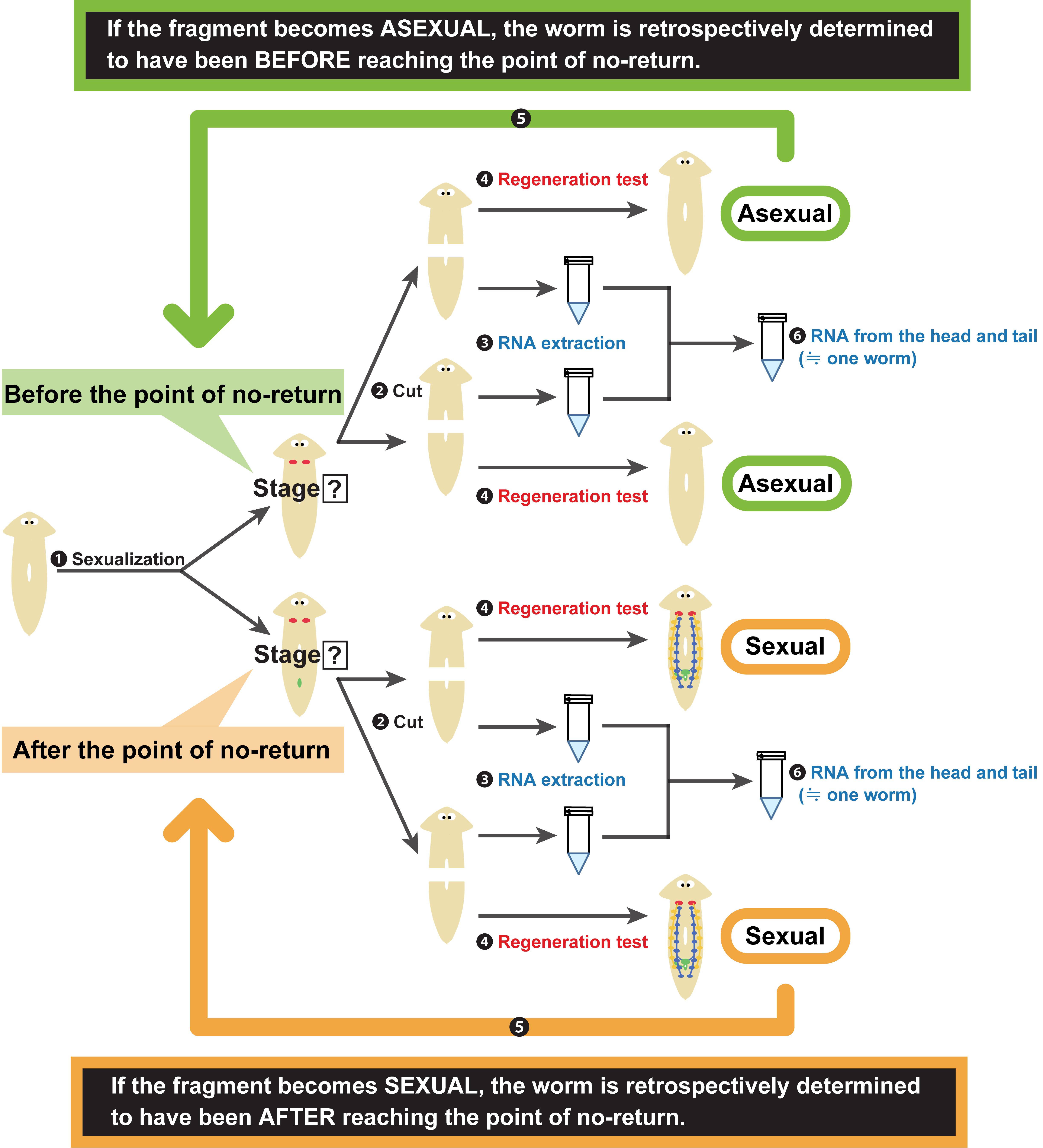

Supplement: S1 Fig — In this study, the samples for RNA-seq library and knockdown experiments were checked using the “regeneration test after sexualization.” Step 1: To sexualize, the test worms (the asexual OH worms) were daily fed with sexually mature worms of Bdellocephala brunnea or the fraction M0 + M10 that contains the sex-inducing substances from B. brunnea during each experimental period. Step 2: After the feeding assay, the test worms were transversely cut into two pieces at the prepharyngeal level. Step 3: Immediately after cutting, total RNAs were extracted from the head fragments of half of the test worms and the tail fragments of the other half. Step 4: Fragments from which RNA was not extracted were allowed to regenerate for approximately 1 month by being fed with beef liver, which does not have sex-inducing activity. Step 5: The reproductive modes of the regenerates were examined. If the test worms have not transgressed the point of no return (e.g., if they are stage 2-worms), both the head- and tail-derived fragments become asexual after regeneration. In contrast, if they have transgressed the point of no return (e.g., if they are stage 3-worms), both the head- and tail-derived fragments become sexual after regeneration. Thus, observation of the reproductive mode of the regenerated fragment can retrospectively determine whether the worms had exceeded or not the point of no return at the time of cutting, for example, even if the morphological features of stage 3 were not developed yet. Step 6: When the reproductive mode of the regenerates was the same, total RNAs extracted from the head and tail fragments of the corresponding other half were mixed. The mixed RNA samples, which were considered as RNAs from one individual, were used for developing the RNA-seq library and RT-qPCR analysis. (TIF) [file pgen.1011944.s001.tif]

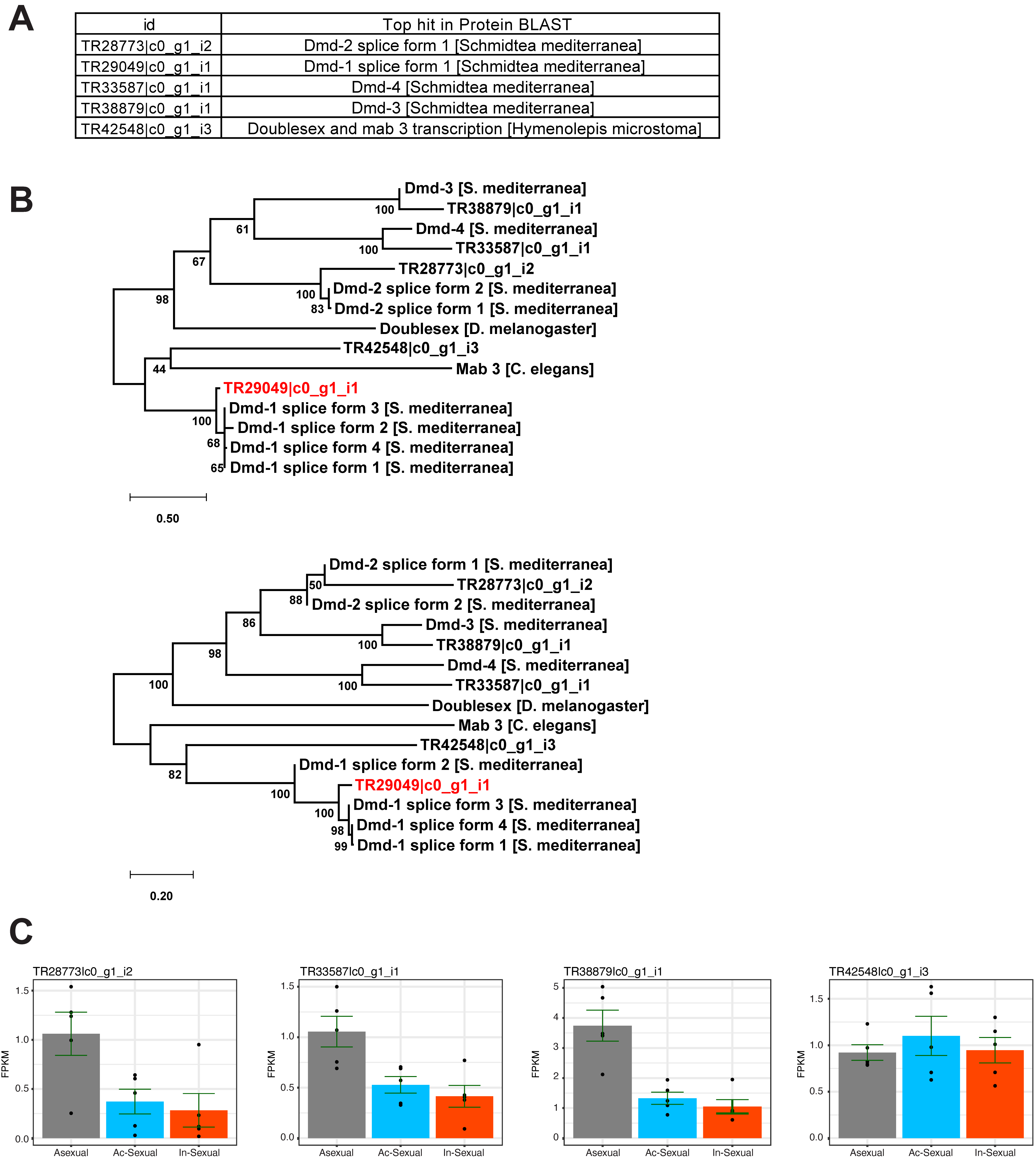

Supplement: S2 Fig — In this study, we named TR29049|c0_g1_i1 Dr-dmd-1. (A) Five genes annotated as double sex and mab3 transcription factor were contained in the transcriptome catalogues of D. ryukyuensis (Sekii et al., 2019). Top hits obtained using Protein BLAST search (https://blast.ncbi.nlm.nih.gov/Blast.cgi) of these DM domain genes as the query are shown. (B) Phylogenic trees for the DM domain genes in D. ryukyuensis using either the maximum likelihood method (upper) or the neighbor-joining method (lower). The percentage of trees in which associated taxa clustered together in the bootstrap (1000 replicates) is shown next to the branches. Scale bars: upper, substitutions per site; lower, evolutionary distance. The maximum likelihood and the neighbor-joining trees were constructed using MEGA version 12.0.11. Accession numbers: Dmd-1 splice form 1, AGL61623; Dmd-1 splice form 2, AGL61624; Dmd-1 splice form 3, AGL61625; Dmd-1 splice form 4, AGL61626; Dmd-2 splice form 1, AGL61627; Dmd-2 splice form 2, AGL61628; Dmd-3, AGL61629; Dmd-4, AGL61630; Doublesex, NP_001262353; Mab 3, NP_001022464. (C) The FPKM values in asexual and sexual worms of the four DM domain genes other than TR29049|c0_g1_i1. Bar graphs were plotted based on fragments per kilobase of exon per million reads mapped (FPKM) value in the RNA-seq data of D. ryukyuensis (Sekii et al., 2019). Two races of sexual worms of D. ryukyuensis occur (Kobayashi et al., 2012). Ac-Sexual: Acquired sexual worms can switch to an asexual state; In-Sexual: Innate sexual worms cannot switch to an asexual state. (TIF) [file pgen.1011944.s002.tif]

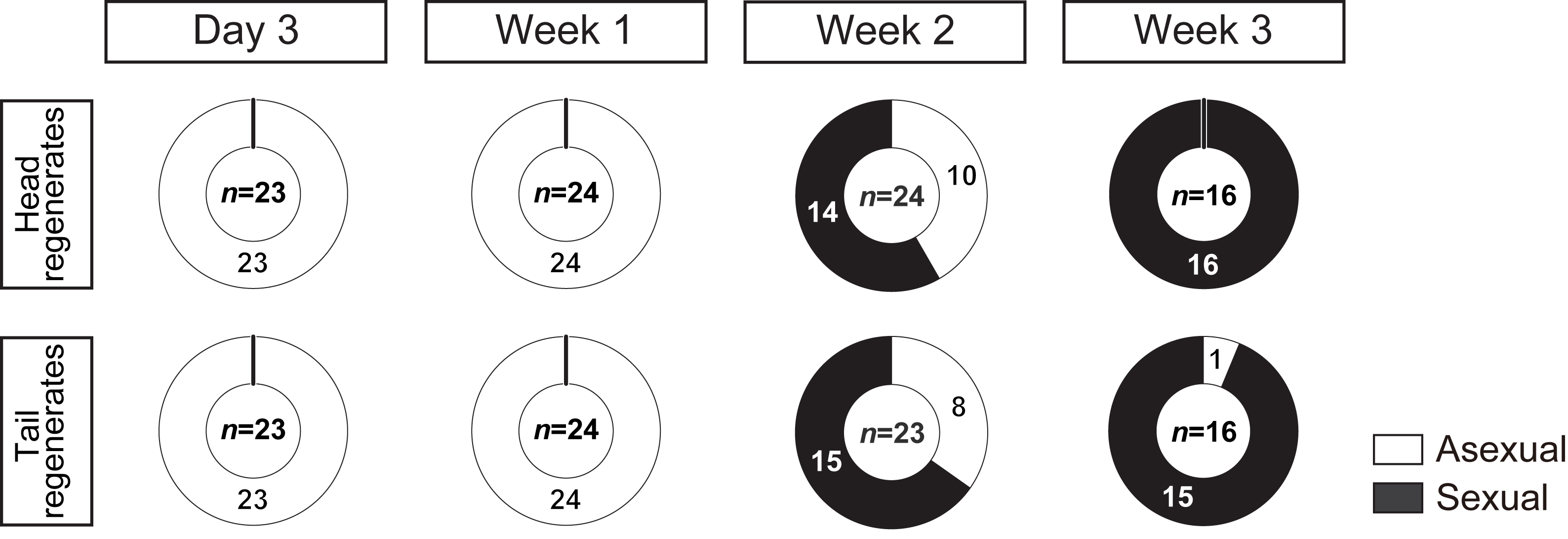

Supplement: S3 Fig — The test worms were fed with minced worms of B. brunnea for 3 d, 1 week, 2 weeks, or 3 weeks. After feeding with minced worms of B. brunnea, the test worm was subjected to a regeneration test shown in S1 Fig. If the fragments become sexual after the regeneration test, it can be determined retrospectively that the worm had exceeded the point of no return at the time of cutting (i.e., after feeding the worm with minced worms of B. brunnea for each duration). Similarly, if the fragments become asexual, it was determined that the worm had not exceeded the point of no return. (TIF) [file pgen.1011944.s003.tif]

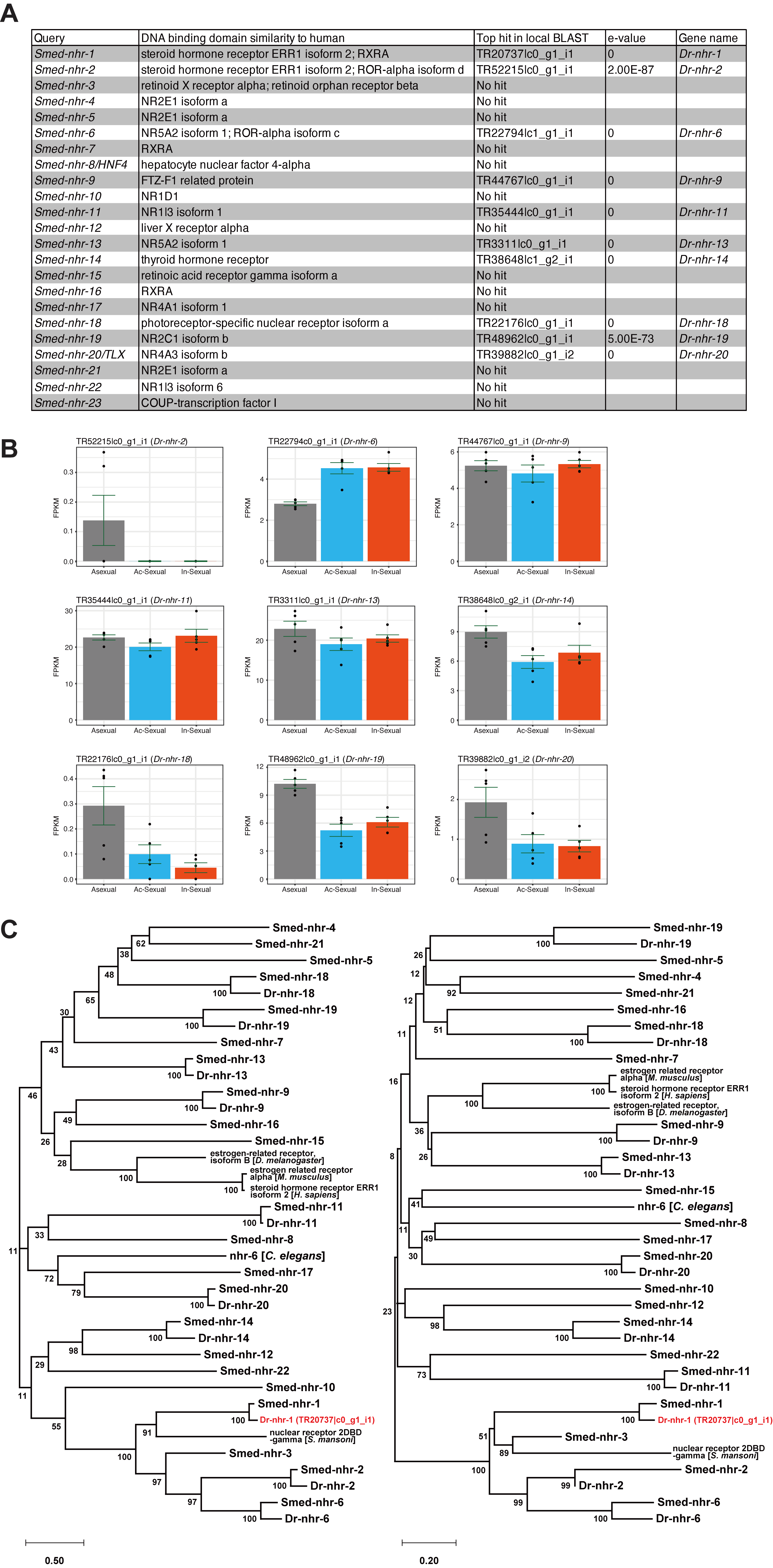

Supplement: S4 Fig — In the current study, we named TR20737|c0_g1_i1 Dr-nhr-1. (A) We performed a local BLASTX search for the transcriptome catalogues of D. ryukyuensis by using 23 nhr genes of S. mediterranea as the query with an e-value cut-off of 10 -30, resulting in ten homologs. The description of the DNA-binding domain similarity to human was reproduced from Table S1 of Tharp et al. (2014). (B) The FPKM values in asexual and sexual worms of the nine nhr genes other than TR20737|c0_g1_i1. Bar graphs were plotted based on FPKM value in the RNA-seq data of D. ryukyuensis (Sekii et al., 2019). Two races of sexual worms of D. ryukyuensis occur (Kobayashi et al., 2012). Ac-Sexual: Acquired sexual worms can switch to an asexual state; In-Sexual: Innate sexual worms cannot switch to an asexual state. (C) Phylogenetic trees for the planarian nhr genes and the nhr genes in other animals using either the maximum likelihood method (left) or the neighbor-joining method (right). The percentage of trees in which associated taxa clustered together in the bootstrap (1000 replicates) is shown next to the branches. Scale bars: left, substitutions per site; right, evolutionary distance. The maximum likelihood and the neighbor-joining trees were constructed using MEGA version 12.0.11. The sequences of 23 nhr genes in S. mediterranea in the supplementary data of Tharp et al. (2014) were used. Accession numbers: estrogen-related receptor alpha [Mus musculus], AAB51250; estrogen-related receptor, isoform B [Drosophila melanogaster], NP_648183; nuclear hormone receptor family member nhr-6 [Caenorhabditis elegans], NP_497731; nuclear receptor 2DBD-gamma [Schistosoma mansoni], AAW88550; steroid hormone receptor ERR1 isoform 2 [Homo sapiens], NP_001269380. (TIF) [file pgen.1011944.s004.tif]

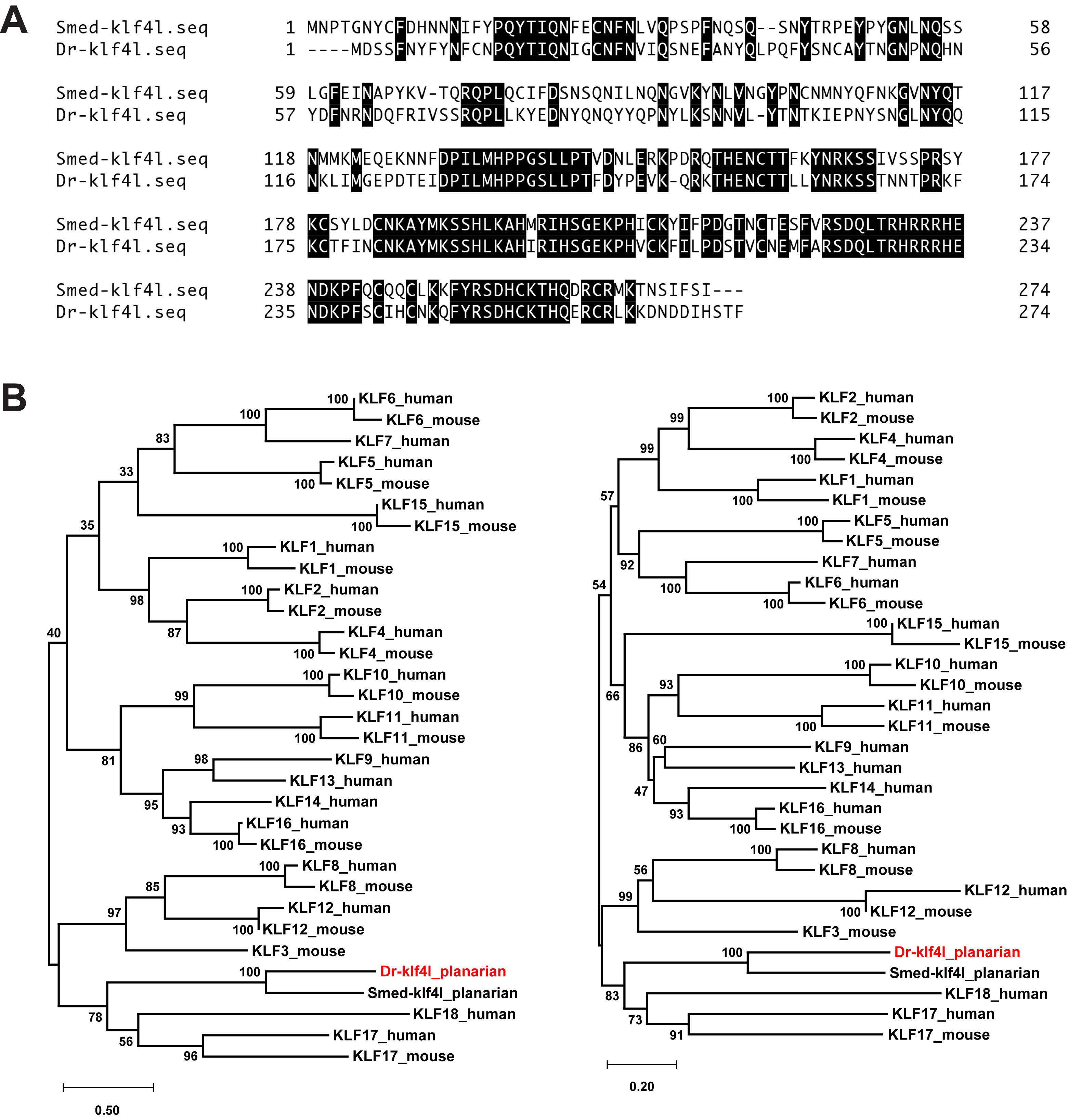

Supplement: S5 Fig — In this study, we named TR16868|c0_g1_i1 Dr-klf4l. (A) We obtained TR16868|c0_g1_i1 as the top hit through a Protein BLAST search of the deduced amino acid sequence of klf4l in S. mediterranea as the query against the transcriptome catalogues of D. ryukyuensis (Sekii et al., 2019). The amino acid sequence homology between Dr-klf4l (TR16868|c0_g1_i1) and klf4l in S. mediterranea was analyzed using the GENETYX-MAC version 13.0.14 (GENETYX, Tokyo, Japan). The amino acid sequence of Dr-klf4l is 54.7% (265 amino acids) identical to that of klf4l in S. mediterranea. The matching amino acids are shaded in black. (B) Phylogenetic trees for the planarian klfl genes and the krüppel-like factor (KLF) family in human and mouse using either the maximum likelihood method (left) or the neighbor-joining method (right). The percentage of trees in which associated taxa clustered together in the bootstrap (1000 replicates) is shown next to the branches. Scale bars: left, substitutions per site; right, evolutionary distance. The maximum likelihood and the neighbor-joining trees were constructed using MEGA version 12.0.11. Accession numbers: KLF1_human, NP_006554; KLF1_mouse, NP_034765; KLF2_human, NP_057354; KLF2_mouse, NP_032478; KLF3_mouse, NP_032479; KLF4_human, NP_001300981; KLF4_mouse, NP_034767; KLF5_human, NP_001721; KLF5_mouse, NP_033899; KLF6_human, NP_001291; KLF6_mouse, NP_035933; KLF7_human, NP_003700; KLF8_human, NP_001311031; KLF8_mouse, NP_001344112; KLF9_human, NP_001197; KLF10_human, NP_001027453; KLF10_mouse, NP_001276400; KLF11_human, NP_001171187; KLF11_mouse, NP_848134; KLF12_human, NP_001387075; KLF12_mouse, NP_001398638; KLF13_human, NP_057079; KLF14_human, NP_619638; KLF15_human, NP_054798; KLF15_mouse, NP_001342597; KLF16_human, NP_114124; KLF16_mouse, NP_510962; KLF17_human, NP_775755; KLF17_mouse, NP_083692; KLF18_human, NP_001345367. (TIF) [file pgen.1011944.s005.tif]

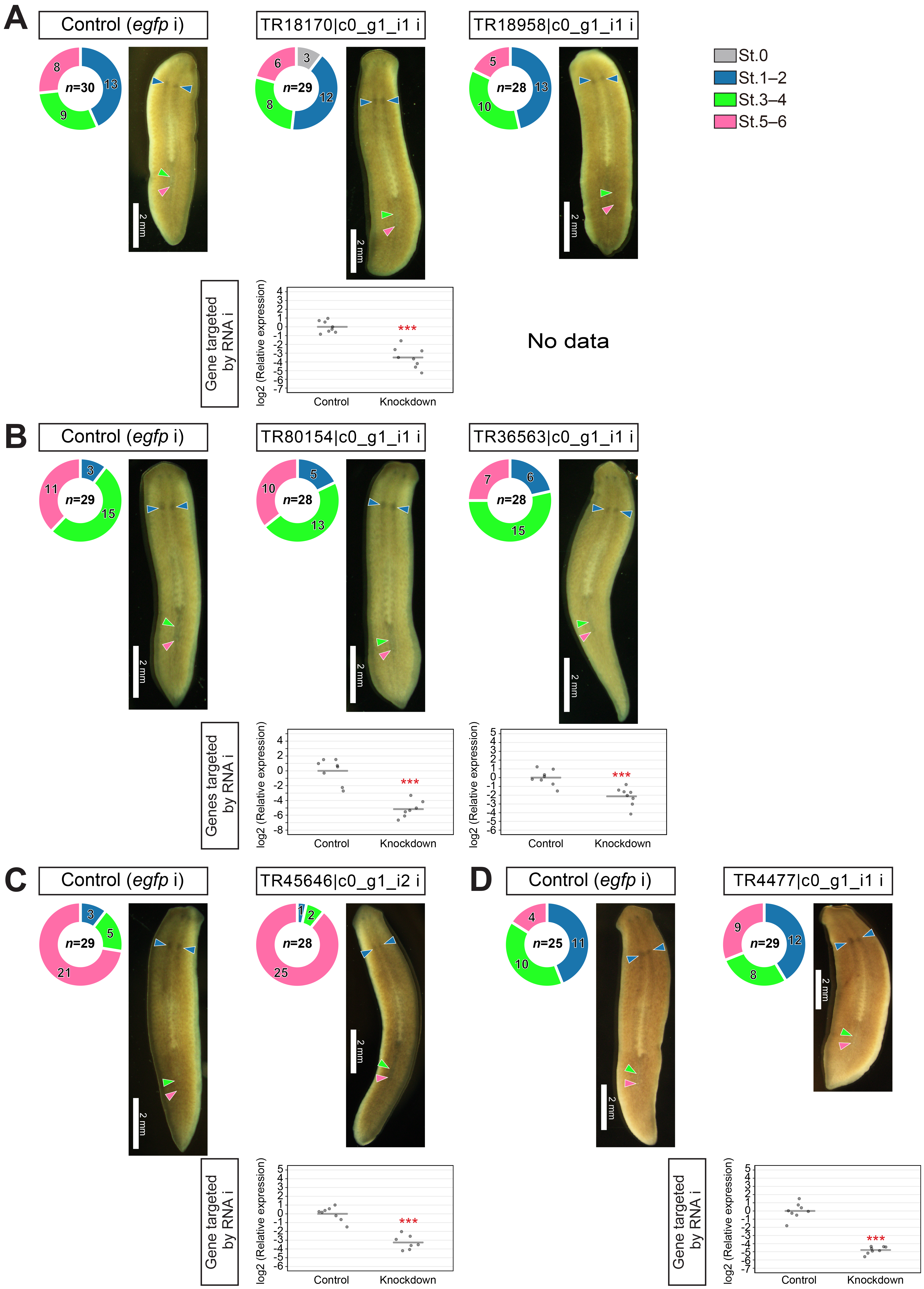

Supplement: S6 Fig — No phenotypes were observed in the knock-down worms of the candidate genes other than Dr-nhr-1, Dr-dmd-1, Dr-klf4l, and TR29311|c0_g1_i1. (A) TR18170|c0_g1_i1 and TR18958|c0_g1_i1. (B) TR80154|c0_g1_i1 and TR36563|c0_g1_i1. (C) TR45646|c0_g1_i2. (D) TR4477|c0_g1_i1. The worms were evaluated through external observation, and the results are shown in a donut chart with four distinctions (Stage 0 [asexual], Stages 1–2, Stages 3–4, and Stages 5–6). The number of test worms after RNAi treatment with sexualization is shown in the center of the doughnut chart. The doughnut chart displays the number of worms at each of the four stages of sexualization in its circular sections. Live ventral images of the most sexually mature test worm are presented. Blue arrowheads highlight a pair of ovaries, while green and pink arrowheads point out the copulatory apparatus and genital pore, respectively. A scale bar, 1 mm. In the knocked-down worms, RNAi efficiency was examined using RT-qPCR. The RT-qPCR data are shown relative to the expression level in the control worm, and log2 (relative expression) on the vertical axis indicates -ΔΔCt. Each circle indicates a control or knocked-down worm. Eight replicates were used, but data were handled as NA (not available) if the expression was in the case of outliers. The bars in the plots indicate the averages of -ΔΔCt. Asterisks indicate significant differences between the control and knocked-down worms (Student’s t-test: ***P < 0.001). Note that the PCR datum for TR18958|c0_g1_i1 was not available because appropriate primers could not be designed. (TIF) [file pgen.1011944.s006.tif]

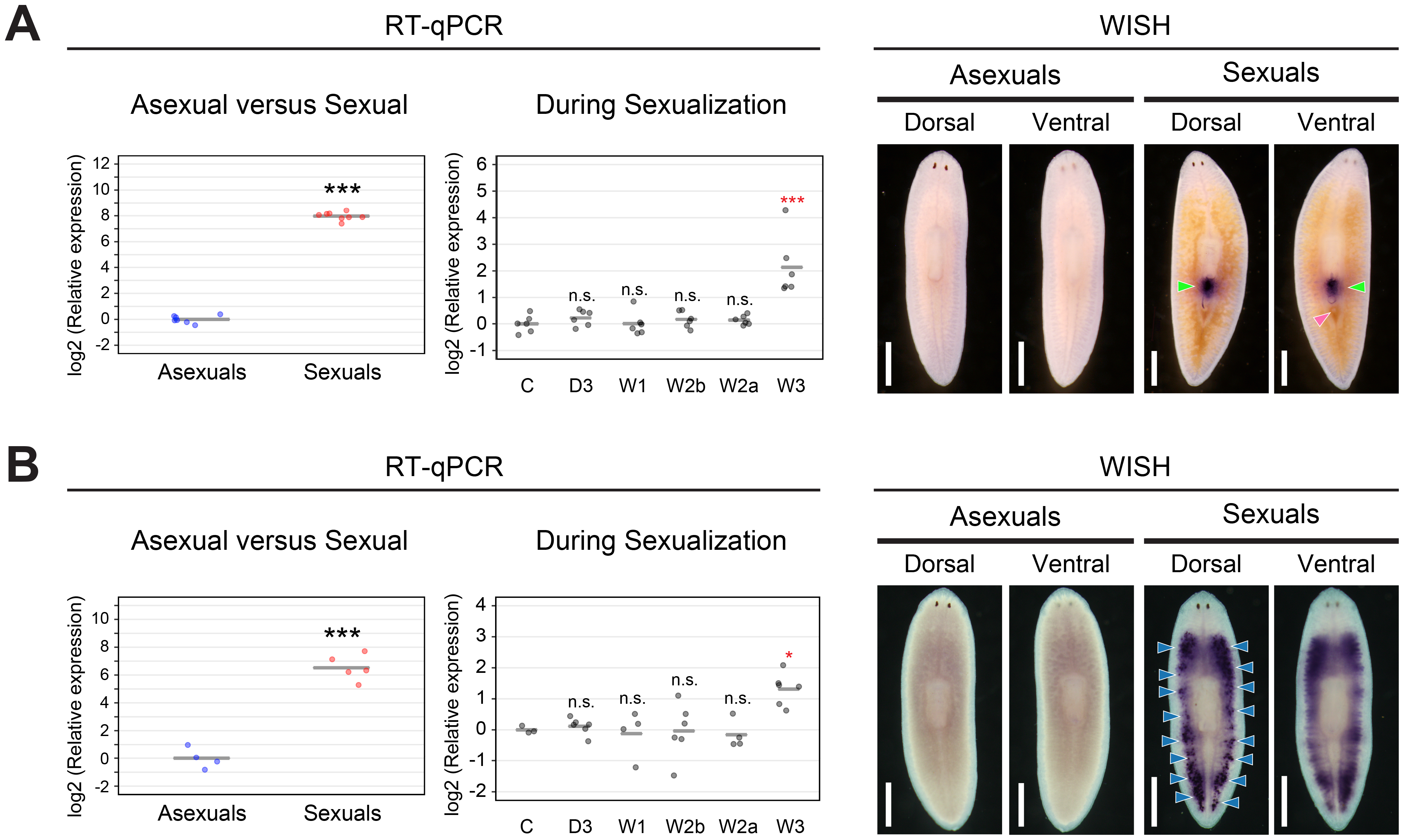

Supplement: S7 Fig — (A) TR44991|c0_g1_i2 was annotated as Cathepsin L. (B) TR34243|c0_g1_i1 was a homolog of C3H-zinc finger-containing protein 1 in S. mediterranea (Wang et al., 2010). (A–B) Expressions in asexual and sexual worms were examined using RT-qPCR analysis. The RT-qPCR data are shown relative to the expression level in the asexual worm, and log2 (relative expression) on the vertical axis indicates -ΔΔCt. Each circle indicates an asexual or a sexual worm. Eight replicates for TR44991|c0_g1_i2 expression and five replicates for TR34243|c0_g1_i1 expression were used, but data were handled as NA (not available) if the expression was too low to be detected. The bars in the plots indicate the averages of -ΔΔCt. Asterisks indicate significant differences between the asexual and sexual worms (Student’s t-test: ***P < 0.001). Changes in expression level during sexualization were also examined using RT-qPCR analysis. C, control worms (asexual OH worms); D3, day 3-worms; W1, week 1-worms; W2b, week 2 before-worms; W2a, week 2 after-worms; W3, week 3-worms. The RT-qPCR data for each candidate essential gene are shown relative to the expression level in the control worm, and log2 (relative expression) on the vertical axis indicates -ΔΔCt. Each circle indicates an individual worm in the control or minced B. brunnea-fed groups. Six replicates were used, but data were handled as NA (not available) if the expression was too low to be detected. The bars in the plots indicate the averages of -ΔΔCt. Asterisks indicate significant differences compared with control worms (Tukey’s HSD test: *P < 0.05; ***P < 0.001; n.s., not significant). Note that these markers started to express around week 3 of sexualization, which was an obvious stage beyond the point of no return (Fig 1B). Representative whole-mount in situ hybridization patterns for the ventral and dorsal sides of worms are shown. The expression pattern was judged based on five and three replicates in the asexual and sexual worms, respectiv [file pgen.1011944.s007.tif]

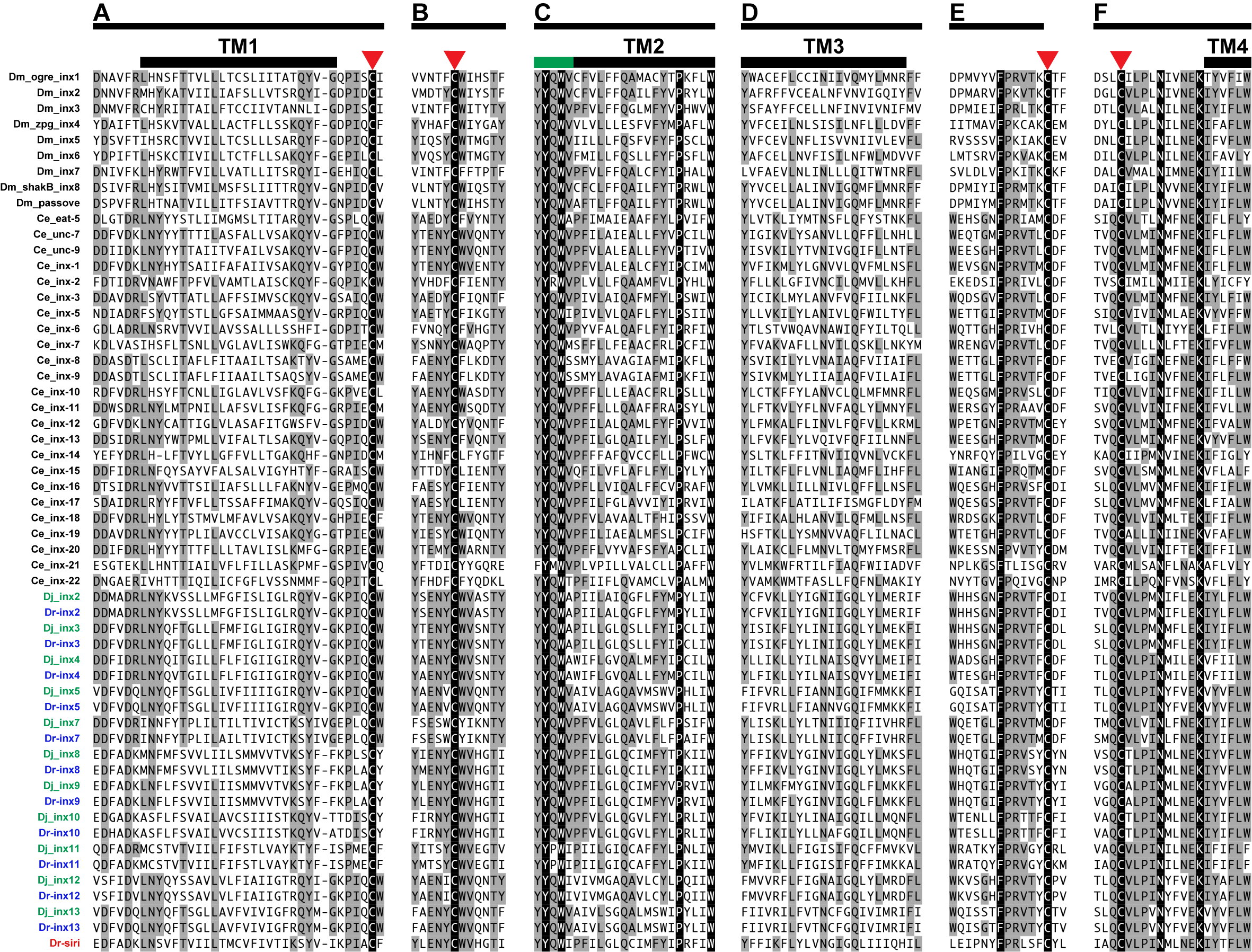

Supplement: S8 Fig — The multiple sequence alignment analysis was performed using the GENETYX-MAC Version 13.0.14 (GENETYX, Tokyo, Japan) according to Nogi and Levin (2005). Sequence alignment of innexin proteins showing highly conserved regions. Black bars indicate the predicted transmembrane domains TM1–TM4. Red arrowheads indicate conserved cysteine residues in the extracellular loops. The matching amino acids are indicated using black shading, and low consensus (≥50%) amino acids are indicated using grey shading. (A) First transmembrane domains and the N-terminal and C-terminal flanking regions. (B) Conserved regions in the first extracellular loops. (C) Second transmembrane domains and the conserved amino acids YYQW(V) at the end of the first extracellular loops. The conserved amino acids are indicated using a green bar. (D) Third transmembrane domains and the C-terminal flanking regions. (E) Conserved regions in the second extracellular loops. (F) A part of the fourth transmembrane domains and the conserved region in the second extracellular loops. Dr-siri (TR37455|c0_g1_i1) in this study is indicated in red, and other innexin genes in D. japonica and D. ryukyuensis are indicated in green and blue, respectively. Dm, Drosophila melanogaster; Ce, Caenorhabditis elegans; Dj, Dugesia japonica; Dr, Dugesia ryukyuensis. Accession numbers: Dm_org_inx1, NP_524824; Dm_inx2, NP_572375; Dm_inx3, NP_524730; Dm_zpg_inx4, NP_648049; Dm_inx5; NP_573353; Dm_inx6, NP_572374; Dm_inx7, NP_788872; Dm_shakB_inx8, NP_728361; Dm_passove, AAA28745; Ce_eat-5, NP_492068, Ce_unc-7, NP_001257255; Ce_unc-9, NP_741917; Ce_inx-1, NP_741826; Ce_inx-2, NP_509885; Ce_inx-3, NP_509002; Ce_inx-5, NP_509403; Ce_inx-6, NP_502435; Ce_inx-7, NP_500894; Ce_inx-8, NP_502209; Ce_inx-9, NP_502210; Ce_inx-10, NP_001024139; Ce_inx-11, NP_001256426; Ce_inx-12, NP_491213; Ce_inx-13, NP_491212; Ce_inx-14, NP_492078; Ce_inx-15, NP_491313; Ce_inx-16, NP_491314; Ce_inx-17, NP_491315; Ce_inx-18, NP_741294; Ce_inx-19, NP_490983; Ce_i [file pgen.1011944.s008.tif]

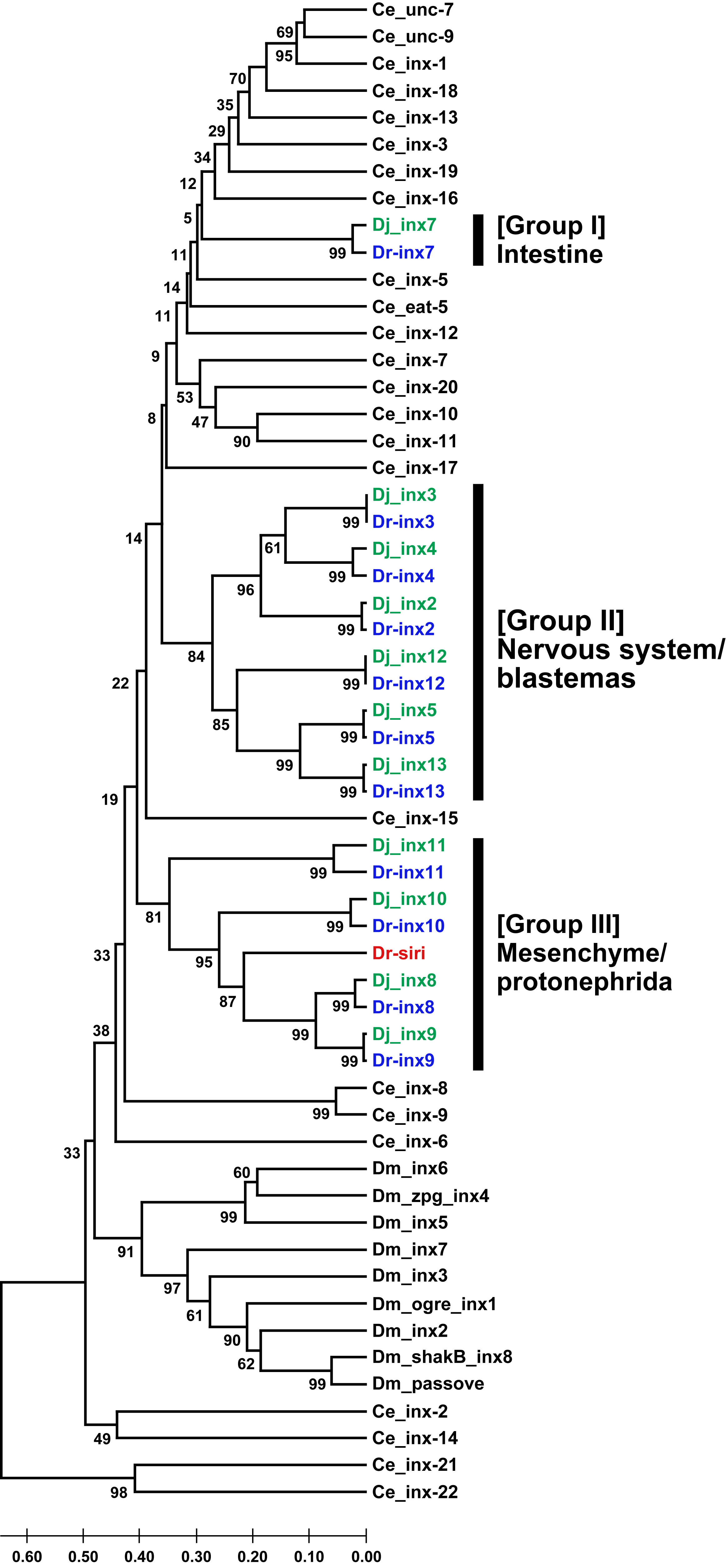

Supplement: S9 Fig — The UPGMA tree was constructed using MEGA version 12.0.11 according to Nogi and Levin (2005). The percentage of trees in which associated taxa clustered together in the bootstrap (1000 replicates) is shown next to the branches. A scale bar: Jaccard similarity coefficient. The predicted amino acid sequences of the conserved region, including the whole of the 1st–4th transmembrane domains (S8A–F Fig), were used for this analysis. Dr-siri (TR37455|c0_g1_i1) in this study is indicated in red, and other innexin genes in D. japonica and D. ryukyuensis are indicated in green and blue, respectively. The three groups of the planarian innexin sequences classified by this analysis (Nogi and Levin, 2005) are indicated by the bars and the names of the groups. Note that Dr-siri (TR37455|c0_g1_i1) was classified into Group III. Dm, Drosophila melanogaster; Ce, Caenorhabditis elegans; Dj, Dugesia japonica; Dr, Dugesia ryukyuensis. Accession numbers: Dm_org_inx1, NP_524824; Dm_inx2, NP_572375; Dm_inx3, NP_524730; Dm_zpg_inx4, NP_648049; Dm_inx5; NP_573353; Dm_inx6, NP_572374; Dm_inx7, NP_788872; Dm_shakB_inx8, NP_728361; Dm_passove, AAA28745; Ce_eat-5, NP_492068, Ce_unc-7, NP_001257255; Ce_unc-9, NP_741917; Ce_inx-1, NP_741826; Ce_inx-2, NP_509885; Ce_inx-3, NP_509002; Ce_inx-5, NP_509403; Ce_inx-6, NP_502435; Ce_inx-7, NP_500894; Ce_inx-8, NP_502209; Ce_inx-9, NP_502210; Ce_inx-10, NP_001024139; Ce_inx-11, NP_001256426; Ce_inx-12, NP_491213; Ce_inx-13, NP_491212; Ce_inx-14, NP_492078; Ce_inx-15, NP_491313; Ce_inx-16, NP_491314; Ce_inx-17, NP_491315; Ce_inx-18, NP_741294; Ce_inx-19, NP_490983; Ce_inx-20, NP_001251236; Ce_inx-21, NP_491187; Ce_inx-22, NP_491186; Dj_inx2, BAE78811; Dj_inx3, BAE78812; Dj_inx4, BAE78813; Dj_inx5, BAE78814; Dj_inx7, BAE78815; Dj_inx8, BAE78816; Dj_inx9, BAE78817; Dj_inx10, BAE78818; Dj_inx11, BAE78819; Dj_inx12, BAD83778 and Dj_inx13, BAE78820. See S7 Table for the information on innexins in Dugesia ryukyuensis. (TIF) [file pgen.1011944.s009.tif]

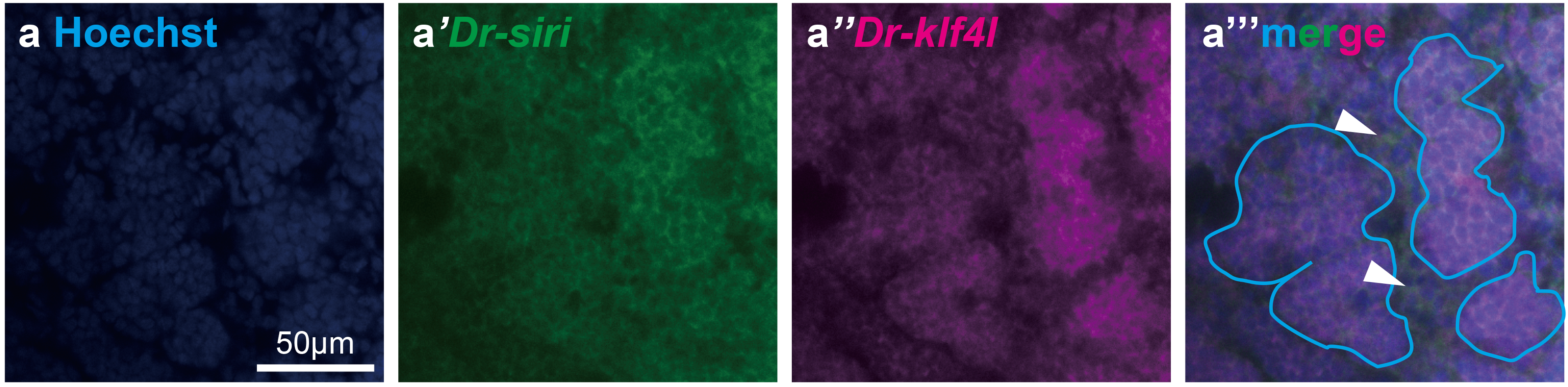

Supplement: S10 Fig — This result of FISH analysis is the low magnification image in Fig 13A. The mesenchymal cells expressing Dr-siri (green) around the testes can be recognized (white arrowheads). (a) The nuclei were counterstained with Hoechst 33342 (blue). (a’) Dr-siri (green), (a”) Dr-klf4l (magenta), and (a”’) the merged images are shown. Domains bound by the blue line are testes. (TIF) [file pgen.1011944.s010.tif]

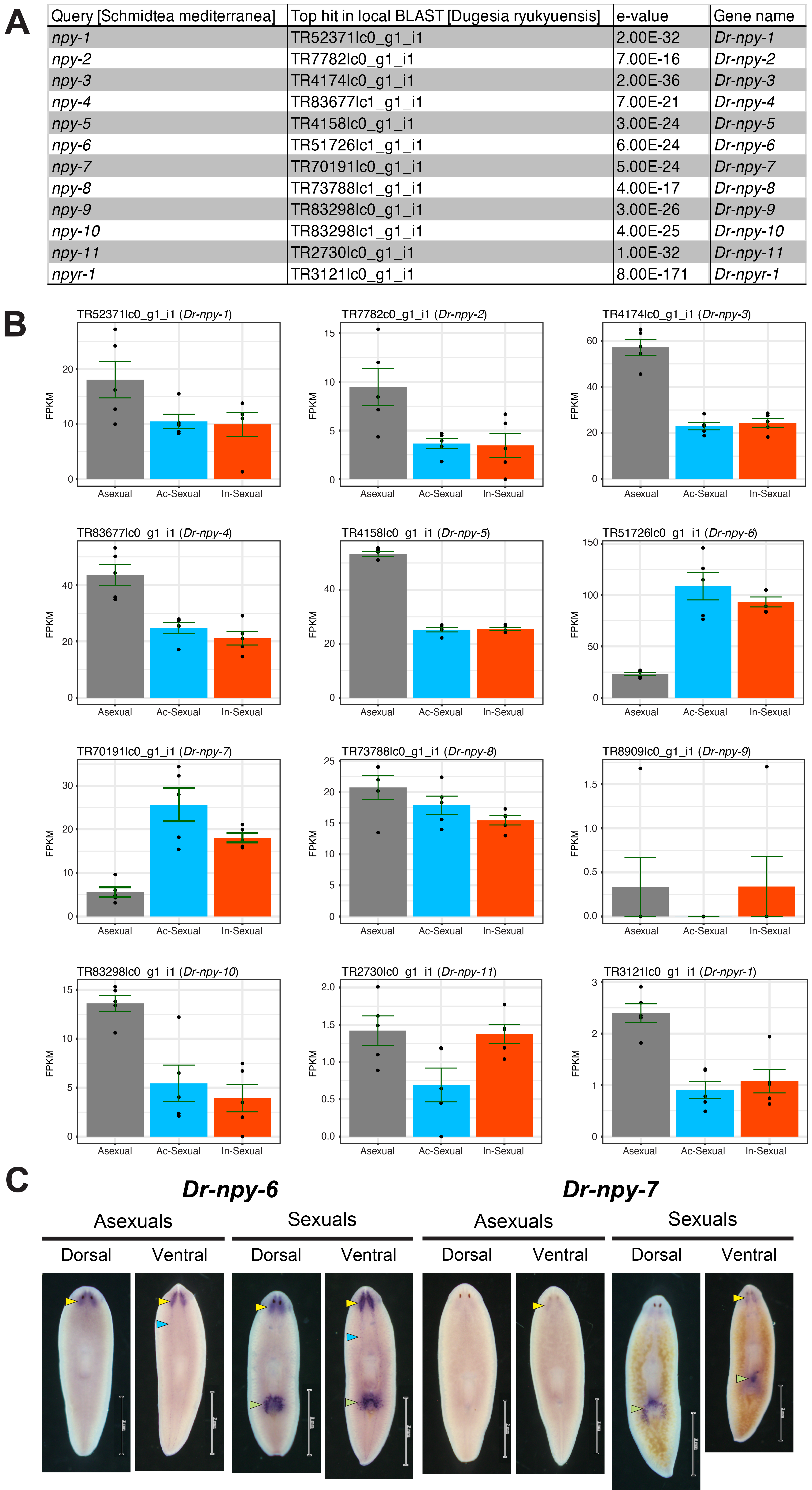

Supplement: S11 Fig — (A) We performed a local BLASTX search for the transcriptome catalogues of D. ryukyuensis (Sekii et al., 2019) by using eleven npy genes and an npyr gene of S. mediterranea. Top hits obtained in the BLAST search are shown. (B) The FPKM values in asexual and sexual worms of the homologs. Bar graphs were plotted based on FPKM value in the RNA-seq data of D. ryukyuensis (Sekii et al., 2019). Two races of sexual worms of D. ryukyuensis occur (Kobayashi et al., 2012). Ac-Sexual: Acquired sexual worms can switch to an asexual state; In-Sexual: Innate sexual worms cannot switch to an asexual state. (C) Whole-mount in situ hybridization patterns for Dr-npy-6 and Dr-npy-7 are shown. The expression pattern was judged based on five and three replicates in the asexual and sexual worms, respectively. Signals were seen as blue/purple staining. Yellow, blue, and green arrowheads indicate a brain, ventral nerve cord, and copulatory apparatus, respectively. A scale bar, 2 mm. (TIF) [file pgen.1011944.s011.tif]
