# Supplementary material for: Identification of an innexin required for termination of the asexual state in planarian reproductive switching
Source: PLoS Genet. 2025 Nov 18;21(11):e1011944. doi: 10.1371/journal.pgen.1011944 (PMC12654950; doi:10.1371/journal.pgen.1011944)
Supplement: Attachment — Submitted filename: PGENETICS-D-25-00534-2_Response_letter_kk.docx [file pgen.1011944.s029.docx]

Dr. Jean-René Huynh, Academic Editor
Dr. Paula Cohen, Section Editor
Dr. Aimée Dudley, Editor-in-Chief
Dr. Anne Goriely, Editor-in-Chief
PLOS Genetics

Dear Sir:

I thank you for allowing us to extend the deadline. The authors thank the two reviewers for their consideration of the manuscript entitled “**Identification of an innexin required for termination of the asexual state in planarian reproductive switching**” (Ms. No. PGENETICS-D-25-00534-2). We have extensively revised the manuscript in response to the in-depth and helpful comments provided by the reviewers.

We believe that the reviewers’ comments have helped greatly to improve the quality of the manuscript. Individual responses to the reviewer comments are given below. Please be advised that the corresponding revisions in the manuscript are highlighted in yellow. We are confident that the revised manuscript will be accepted in “PLOS Genetics.”

Sincerely,

Dr. Kazuya Kobayashi

Professor

Faculty of Agriculture and Life Science

Hirosaki University

3 Bunkyo-cho, Hirosaki, Aomori 036-8561, Japan

E-mail: kobkyram@hirosaki-u.ac.jp

Tel/Fax: +81-172-39-3587

Point-by-point reply to the reviewers

We sincerely thank the reviewers for their valuable feedback on the manuscript. We have addressed all the comments provided and incorporated them to improve the manuscript. Please be advised that the corresponding revisions in the MS are highlighted in yellow.

Reviewer 1

（１）Line 468: The authors state that Dr-nanos expression in Dr-dmd-1(RNAi) animals is similar to controls, but significantly reduced in both Dr-nhr-1(RNAi) and Dr-klf4l(RNAi) animals. They also show that Dr-klf4l does not differ significantly in Dr-dmd-1(RNAi) animals (Figure 8). However, if no testes are observable in Dr-dmd-1(RNAi) animals (line 440), why isn’t the expression of Dr-nanos and Dr-klf4l reduced in these animals, given that they both are expressed in testis germ cells?

Thank you for pointing that out. We had the same question as Reviewer 1, so we performed WISH to examine the expression of *Dr-nanos*, *Dr-klf4l*, and *Dr-nhr-1* in the knocked-down worms of *Dr-dmd-1*. We added this result to Fig 8 and incorporated the following explanation and discussion into the MS: Line 477–490 “To understand why *Dr-nanos* and *Dr-klf4l* expression, which are expressed in the testes, did not decrease in *Dr-dmd-1* (RNAi) worms despite the lack of testicular differentiation, we performed WISH analysis of *Dr-nanos*, *Dr-klf4l,* and *Dr-nhr-1* in *Dr-dmd-1* (RNAi) worms (Fig 8E). There was an important difference in the *Dr-nanos* and *Dr-klf4l* expression patterns in the dorsal regions of the control and the knocked-down worms. In the control worms, *Dr-nanos* and *Dr-klf4l* were expressed in the testes (Fig 8E, blue arrowheads), whereas in the knocked-down worms, dot-like expression of *Dr-nanos* and *Dr-klf4l* was detected around the prepharyngeal region (Fig 8E, white arrowheads). Although testes have not been formed in *Dr-dmd-1* (RNAi) worms (Figs 5 and 6), *Dr-klf4l + / Dr-nanos +* cells appear to have been induced. The *Dr-nanos* and *Dr-klf4l* expression patterns in the ovaries and supernumerary ovaries were the same in the ventral regions of the control and the knocked-down worms. There was no difference in the expression pattern of *Dr-nhr-1* between the control and the knocked-down worms.”

(2) The FISH images in Figure 13 are unclear and should be repeated. The figure does not convincingly show that Dr-siri is expressed in both Dr-kl4l, Dr-nanos, and Dr-dmd-1-expressing cells (i.e. in both germ cells and testis niche cells). At a minimum, the authors should show that their FISH method can clearly distinguish between the expression of Dr-nanos/klf4l and Dr-dmd-1, which should not appear to co-express, before drawing strong conclusions about where Dr-siri is expressed using this technique. Either way, it is not necessary for Dr-siri to be expressed in both germ cells and niche cells (for gap junction formation between these cells) for the model to be correct. Even if it is only expressed in one cell type in the testis, other innexins could be expressed in the other cell type to facilitate gap junctional communication.

Compared to *S. mediterranea*, for which whole-mount FISH analysis is frequently performed, *D. ryukyuensis* OH worms (especially sexual worms) are relatively large and thick, and tend to yield poor results with whole-mount FISH method. The whole-mount FISH results shown in original Fig 13 represent the best results we could achieve. Therefore, following this Reviewer 1's comment, we performed paraffin section FISH (Lines 747–789; new Fig 13). We clearly demonstrated the colocalization of *Dr-nanos* and *Dr-klf4l* in cells within the testis (Fig 13A). However, *Dr-dmd-1* expression was very low. In the double staining using FISH on *Dr-nanos* and *Dr-dmd-1*, no cells were observed in which the signals from the two genes coincided, though it was a rare occurrence (Fig 13E). We present the original Fig 13 as the new Fig 14 in the revised MS. In the original Fig 13, we had concluded that there were cells expressing *Dr-dmd-1*, but in the new Fig 14, we changed our assertion to state that no clear expression of *Dr-dmd-1* was observed. However, fortunately, paraffin section FISH analysis on *Dr-nanos*, *Dr-klf4l,* and *Dr-siri* revealed that not only cells in which *Dr-nanos* and *Dr-siri*, or *Dr-klf4l* and *Dr-siri* co-expressed, but also cells in which *Dr-siri* was expressed alone exist in the testis (Fig 13B, C).

Based on these results, we made the following additions and modifications to the text in the DISCUSSION section. Please note that we have slightly modified the model proposed in this study in the new Fig 17. We defined some of the cells expressing *Dr-dmd-1* in the presumptive differentiation region of testes during sexual induction as precursor cells of niche cells, and cells expressing *Dr-siri* alone in the mature testis as niche cells. Furthermore, we defined cells expressing *Dr-klf4l* and *Dr-nanos* in the presumptive differentiation region of testes during sexual induction as precursor cells of GSCs, and cells expressing *Dr-siri*, *Dr-nanos*, and *Dr-klf4l* in mature testes as GSCs.

Line 982–984: Paraffin section FISH analysis revealed that there were *Dr-klf4l + / Dr-nanos +* cells and *Dr-klf4l – / Dr-nanos +* cells in the testes of *D. ryukyuensis* (Fig 13A).

Line 992–996: These results suggest that, in the presumptive differentiation region of testes during sexual induction, *Dr-dmd-1,* expressed in the precursor cells of niche cells, and *Dr-klf4l* and *Dr-nanos,* expressed in the precursor cells of GSCs, are independently involved in testicular differentiation downstream of *Dr-nhr-1* (Fig 17).

Line 1023–1032: In this study, it was shown that *Dr-siri* was co-expressed with *Dr-klf4l* and *Dr-nanos*, which are expressed in germline cells, including GSCs in the planarian testis [52, 55, 63, 64] (Figs 13B, C and 14A, B; yellow arrowheads). Contrary to our expectations, *Dr-dmd-1*, which was expected to be expressed in somatic niche cells, was scarcely detected in the testes of mature sexually mature worms (Figs 13D–E and 14C). However, paraffin section FISH analysis on *Dr-nanos* or *Dr-klf4l* and *Dr-siri* revealed that not only cells in which *Dr-nanos* or *Dr-klf4l* and *Dr-siri* co-expressed, but also cells in which *Dr-siri* was expressed alone exist in the testis (Fig 13B, C). We suggest that the cells expressing *Dr-siri* alone in the testes of *D. ryukyuensis* are somatic cells, including niche cells.

Line 1056–1065: Paraffin section and whole-mount FISH analyses of sexual worms revealed that *Dr-klf4l* and *Dr-nanos* were co-expressed, and *Dr-siri* was co-expressed with *Dr-klf4l* or *Dr-nanos* in the testis (Figs 13A–C and 14A, B; yellow arrowheads). We expect that the precursor cells of GSCs in which *Dr-klf4l* and *Dr-nanos* expressed may express *Dr-siri* to differentiate into mature GSCs in testes (Fig 17). Furthermore, we found cells expressing *Dr-siri* alone in the testis of sexually mature worms (Fig 13B, C; white arrowheads), though cells expressing *Dr-dmd-1* were hardly recognized (Figs 13D–F and 14C). We expect that the precursor cells of niche cells in which *Dr-dmd-1* expressed may disappear *Dr-dmd-1* and instead express *Dr-siri* to differentiate into mature niche cells (Fig 17).

Reviewr 2

(1) It is important to be sure of the identity of each of these genes when assigning names and determining homology. Neighbour Joining methods are quick but not sufficiently robust and reliable, and a maximum likelihood approach should be used in addition to the neighbour joining trees (the software IQ-Tree, for example, has good documentation and offers model finding). Additionally, it is important where possible to include validated genes from multiple species in addition to Schmidtea mediterranea, C. elegans etc. Please also include bootstraps values on your trees.

Per your recommendation, we performed maximum likelihood and neighbor-joining analyses with bootstrap values on *Dr-dmd-1* (S2 Fig), *Dr-nhr-1* (S4 Fig), and *Dr-klf4l* (S5 Fig) using MEGA version 12.0.11. Since Nogi and Levin (2005) conducted a molecular phylogenetic analysis of planarian innexin gene using the UPGMA method, we performed the UPGMA method for *Dr-siri* in this MS. We reanalyzed it with bootstrap values and Jaccard similarity coefficient using MEGA version 12.0.11 (S9 Fig).

(2) The authors conclude that masculinization is crucial for switching from asexual to sexual reproduction, however I think this is an overstatement and do not think the results conclusively show this as they do not test the alternative. For example, what happens if genes involved in ovary/oocyte differentiation are knocked down such that testes develop normally? The discussion should be reworded accodingly, with the addition of "might" for example.

As Reviewer 2 pointed out, we also determined that the use of the technical term “masculinization” was inappropriate, so we removed it entirely and replaced it with “the production of testes” or “testes production”. Furthermore, we used the expression “might” in the relevant sentence.

(3) S1 Fig. This figure helps a lot to clarify the approach. However, if I understood correctly, half the head and half the tail fragments are left to regenerate, and the respective other halves are used to extract RNA. Once the sexual state is determined, then the RNA extractions are labelled as either asexual or sexual and pooled with RNA samples of the same category. The figure only shows heads, which is a bit confusing, and I think it could help to add tails to this figure.

The original image of S1 Fig does not confuse Reviewer 2. When we created a combined and compressed image for submission, strangely enough, the tail have been lost. We have reposted the correct image of S1 Fig, so please check it.

(4) Fig 6 the top row label was confusing at first and the figure legend did not help. To help guide the reader, you could say explicitly that the top row refects expression of the gene targeted by RNAi respectively.

As Reviewer 2 pointed out, we changed the top row label in Fig. 6 to “Genes targeted by RNAi.” We also added the following to the legend: “ The top row shows the RNAi efficiency of *Dr-nhr-1,* *Dr-dmd-1,* and *Dr-klf4l*, respectively. The bottom three rows show the differentiation levels of the ovaries, testes, vitellaria, and copulatory apparatus in the knocked-down worms of *Dr-nhr-1,* *Dr-dmd-1,* and *Dr-klf4l*.” In connection with this, we have modified the same labels and legends in Fig. 12 and S6 Fig.

(5) line 366 "RNA treatment was stated" perhaps this is a typo for started. 'Initiated with' could be an appropriate substitute.

Thank you for pointing that out. We have corrected it.

(6) Line 366 The experimental approach is not completely clear to me. The worms are cut into three pieces, what happens to the other pieces (non pharangeal)? Are these discarded?

As Reviewer 2 pointed out, the other pieces were not used in the knockdown experiment. We added the following explanation: Line 374–377 “The head fragments and tail fragments without pharynx were not used in the feeding experiments, because they took longer to regenerate to the point where feeding was possible compared to the middle fragments.”

And line 376, just to be sure that you mean that half is left to regenerate and half are taken for RNA extraction.

This is as Reviewer 2 pointed out. Additionally, some were used for histological examination. We have improved the text by changing the order of explanations in lines 384–419 to make it easier for readers to understand.

(7) line 459 "No phenotypes were observed in the knockdown other than Dr-nhr-1, Dr-dmd-1, Dr-klf4l, and TR29311|c0_g1_i1 (S7 Fig), RNAi of which died because of curling and lysis phenotypes, regardless of sexualization." I do not understand this sentence. Dr-nhr-1, Dr-dmd-1, and Dr-klf4l are genes, not phenotypes. I also cannot understand the statement that knockdown of these genes led to death, given the description of the phenotypes in the previous two paragraphs. I also have trouble understanding S7 Fig and its relation to this statement. The figure legend does not seem to match the figure. Is this the correct figure legend?

Per your recommendation, we improved the text as follows: Line 463–466 “The knocked-down worms of TR29311|c0_g1_i1 died due to curling and lysis phenotypes, regardless of sexualization. No phenotypes were observed in the knock-down worms of the candidate genes other than *Dr-nhr-1,* *Dr-dmd-1*, *Dr-klf4l*, and TR29311|c0_g1_i1 (S6 Fig).” As Reviewer 2 pointed out, we had incorrectly labeled the figure and its legend. We have corrected this error.
